# The *BIRC* Family Genes Expression in Patients with Triple Negative Breast Cancer

**DOI:** 10.3390/ijms22041820

**Published:** 2021-02-12

**Authors:** Anna Makuch-Kocka, Janusz Kocki, Anna Brzozowska, Jacek Bogucki, Przemysław Kołodziej, Bartosz J. Płachno, Anna Bogucka-Kocka

**Affiliations:** 1 Department of Pharmacology, Medical University of Lublin, 4a Chodźki St., 20-093 Lublin, Poland; 2 Chair of Medical Genetics, Department of Clinical Genetics, Medical University of Lublin, 11 Radziwiłłowska St., 20-400 Lublin, Poland; janusz.kocki@umlub.pl; 3 Department of Radiotherapy, St. John of Dukla Lublin Region Cancer Center, 20-090 Lublin, Poland; annbrzo@poczta.onet.pl; 4 Department of Organic Chemistry, Medical University of Lublin, 4A Chodźki St., 20-093 Lublin, Poland; jacek.bogucki@umlub.pl; 5 Chair and Department of Biology and Genetics, Medical University of Lublin, 4a Chodźki St., 20-093 Lublin, Poland; przemyslaw.kolodziej@umlub.pl (P.K.); anna.bogucka-kocka@umlub.pl (A.B.-K.); 6 Department of Plant Cytology and Embryology, Institute of Botany, Faculty of Biology, Jagiellonian University in Kraków, 9 Gronostajowa St., 30-387 Kraków, Poland; bartosz.plachno@uj.edu.pl

**Keywords:** triple negative breast cancer, inhibitors of apoptosis proteins, gene expression regulation, BIRC family genes

## Abstract

The *BIRC* (baculoviral IAP repeat-containing; BIRC) family genes encode for Inhibitor of Apoptosis (IAP) proteins. The dysregulation of the expression levels of the genes in question in cancer tissue as compared to normal tissue suggests that the apoptosis process in cancer cells was disturbed, which may be associated with the development and chemoresistance of triple negative breast cancer (TNBC). In our study, we determined the expression level of eight genes from the BIRC family using the Real-Time PCR method in patients with TNBC and compared the obtained results with clinical data. Additionally, using bioinformatics tools (Ualcan and The Breast Cancer Gene-Expression Miner v4.5 (bc-GenExMiner v4.5)), we compared our data with the data in the Cancer Genome Atlas (TCGA) database. We observed diverse expression pattern among the studied genes in breast cancer tissue. Comparing the expression level of the studied genes with the clinical data, we found that in patients diagnosed with breast cancer under the age of 50, the expression levels of all studied genes were higher compared to patients diagnosed after the age of 50. We observed that in patients with invasion of neoplastic cells into lymphatic vessels and fat tissue, the expression levels of *BIRC* family genes were lower compared to patients in whom these features were not noted. Statistically significant differences in gene expression were also noted in patients classified into three groups depending on the basis of the Scarff-Bloom and Richardson (SBR) Grading System.

## 1. Introduction

Breast cancer (BC) is the most commonly diagnosed cancer among the female population. Every year, approximately 2.4 million new cases of breast cancer are diagnosed in the world, and more than 523,000 people die from this cancer [1]. BC is a highly heterogeneous cancer with respect to its molecular, histopathological, and clinical characteristics as well as its treatment and prognosis. It is essential to identify the cancer subtype before starting treatment. Patient survival results vary depending on the BC subtype. The molecular subtype of BC is associated with expression of progesterone receptor (PR), estrogen receptor (ER), and human epidermal growth factor receptor 2 (HER2) [2]. 

Patients with triple negative breast cancer (TNBC) do not express the ER receptor, PR and HER2. TNBC is the BC subtype that is most aggressive and invasive. It accounts for approximately 15–20% of all breast cancer cases [3]. In comparison with other types of breast cancer, TNBC shows unfavorable prognostic features: increased frequency of visceral metastases, shorter interval without recurrence, and higher nuclear grade [4,5]. The problem of poor prognosis in patients with TNBC results from the limitations of the choice of treatment. Currently, treatment for TNBC consists mainly of doxorubicin, paclitaxel, cyclophosphamide, and immunotherapy [6,7]. There are no specific molecular targets in TNBC to underpin targeted therapy and one of the reasons for the failure of the applied pharmacotherapy is the inhibition of tumor cell apoptosis [8,9,10,11].

According to the literature data, proteins from the family of apoptosis inhibitors (IAP), encoded from the *BIRC* (baculoviral IAP repeat-containing; BIRC) family genes, play a key role in developing resistance to apoptosis by various cancer cells (including the breast cancer cells) [12,13]. Eight genes of the *BIRC* family encoding the following IAP proteins have been identified thus far: NLRB/BIRC1/NAIP; BIRC2/human IAP2/cellular IAP1/cIAP1; BIRC3/human IAP1/cell IAP2, cIAP2; XIAP/BIRC4; BIRC5/survivin; BIRC6/BRUCE/APOLLON; BIRC7/livin/KIAP/ML-IAP and IAP/BIRC8/hILP-2 specific for the testis/Ts-IAP [13,14,15].

IAPs form a group of proteins with high structural and functional similarity. These proteins have two unique characteristics: they are the only cellular factors that target executive and initiating caspases, their effects can vary considerably from antiapoptotic to proapoptotic [16]. IAPs also influence other cellular processes: cell cycle, immune system, gene translation and transcription, repair of DNA damage, and signal transduction [15]. They indirectly participate in the signaling pathway of the nuclear factor kappa B (NF-κB) and interfere the proapoptotic secondary mitochondria-derived activator of caspases/direct inhibitor of apoptosis-binding protein with low pI (SMAC/DIABLO) signaling [17,18].

One of the representatives of the genes in the *BIRC* family is *BIRC1* (Baculoviral IAP Repeat-Containing Protein 1) gene. The *BIRC1* gene is located on chromosome 5q13 and is part of a 500kb inverted duplication. The *BIRC1* gene encodes the neuronal apoptosis inhibiting protein (NAIP) and it is a modifier of spinal muscular atrophy resulting from a mutation in a neighboring *SMN1* (Survival of Motor Neuron 1) gene [14]. The role of the *BIRC1* gene and the NAIP protein in cancers is not fully understood.

The *BIRC2* (Baculoviral IAP Repeat Containing 2) and *BIRC3* (Baculoviral IAP Repeat Containing 3) genes encode the Cellular Inhibitor of Apoptosis Protein-1 (cIAP1) and Cellular Inhibitor of Apoptosis Protein-2 (cIAP2) proteins, respectively [14]. The cIAP1 protein participates in the regulation of apoptosis through interaction with caspases. It helps in inflammatory as well as mitogen kinase signaling, immunity, cell proliferation and invasion, metastasis. The cIAP1, through E3 ubiquitin–protein ligase, regulates canonical and noncanonical pathway NF-kappa-B signaling. For the canonical NF-kappa-B signaling pathway, cIAP1 is a constructive positive regulator while for noncanonical pathway is a constitutive suppressor [14]. The cIAP1 and cIAP2 proteins can activate the NFκB pathway also through degradation of IκB inhibitors. The cIAP proteins also have an effect on the Tumor necrosis factor receptor 1 (TNFR1) receptors, that mediate the activation of NFκB. Most likely, cIAP proteins participate in the Wnt signaling pathway and protect cells against death by regulating the activity of protein kinases interacting with receptor 1 and 3 (RIPK) [19,20,21,22]. These proteins also regulate the death process of the ripoptosome, necrosome, and inflammasome [23,24,25,26,27]. The *BIRC2* gene is overexpressed in radiation-resistant tumor cells [28].

The *BIRC4* (Baculoviral IAP Repeat Containing 4) gene encodes the XIAP (X-Linked Inhibitor of Apoptosis) protein—a potent apoptosis suppressor protein, binding to caspase 3 and 7 that can inhibit cell death proteases. It is responsible for the inhibition of the enzymatic activity of caspases and apoptosis as a result of binding to TNF receptor-associated factor 1 (TRAF1) and TRAF2. It is also involved in cell division and metastasis [14].

A functionally similar protein to XIAP is survivin, encoded by the *BIRC5* (Baculoviral IAP Repeat Containing 5) gene. Normally, survivin is expressed during embryonic development. It is presence has been demonstrated in fetal organs, including in the kidneys, brain, liver, lungs, and digestive tract. In a mature organism, this protein is present in small amounts in tissues with a high proliferation potential, undergoing constant renewal (placenta, endometrium, CD34 + stem cells). Survivin expression is not found in normal, differentiated tissues of adults [14]. This protein is expressed in cancer cells. Survivin has been found in cells of almost all types of cancer, including breast cancer, prostate cancer, colorectal cancer, lung cancer, pancreatic cancer, liver cancer, lymphoma, glioblastoma cancer. Overexpression of *BIRC4* and *BIRC5* genes has been described in many cancers, and high expression of survivin and XIAP was associated with poor prognosis [29,30,31,32,33]. Survivin is expressed in large quantity in cancer tissue including breast cancer [34]. High levels of survivin in patients with breast cancer are associated with poor prognosis and resistance to chemotherapy [35,36,37,38].

Another protein encoded by genes from the *BIRC* family that plays a significant role in cancer is APOLLON. APOLLON protein encoded by the *BIRC6* (Baculoviral IAP Repeat Containing 6) gene has the ability to inhibit the caspase cascade and consequently, apoptosis [39], but it also plays a cytoprotective role and regulates cytokinesis [40,41]. APOLLON participates in the process of developing resistance by cells to damaging stimuli [39]. This protein participates in the proteasomal degradation of proapoptotic proteins, including SMAC/DIABLO, caspase 9, and Serine protease HTRA2, mitochondrial (HTRA2/OMI) [42,43]. APOLLON protein overexpression is observed in melanoma, nonsmall cell lung cancer, prostate cancer, and colorectal cancer [39,44,45,46]. 

An important role in cancers is played by livina—a protein encoded by the *BIRC7* (Baculoviral IAP Repeat Containing 5) gene. The *BIRC7* gene encodes two splicing variants (livin α and livin β) [47]. Livin α and livin β show different antiapoptotic effects in vitro. Livin α is associated with cell resistance to staurosporin, while livin β induces cell resistance to TNF-α-induced apoptosis, UV radiation, and etoposide [47,48]. Livin has an antiapoptotic effect—it inhibits caspases 3, 7, and 9 and Smac/DIABLO [49]. Livin activates AKT signaling, promotes tumor progression, and is also involved in inducing trastuzumab resistance in breast cancer [50,51]. Livin overexpression is also seen in nonsmall cell lung cancer, bladder and colon cancer, hepatocellular carcinoma, adrenocortical tumors, and germ cell cancer [52,53,54,55,56]. Livin overexpression is usually associated with the resistance of cancer cells to pharmacotherapy and cancer progression. It has also been found that downregulation of livin expression may result in resensitization of cells to chemotherapy and apoptosis [57].

Apoptosis inhibitor proteins and genes from the *BIRC* family not only control cell death, but also affect the signals of communication pathways, therefore an extremely important aspect of further research on the described genes and proteins is their potential use as a target of new strategies for targeted anticancer therapy. In order to understand the specific role of the *BIRC* family genes in cancers, it is important to accurately determine the expression level of these genes in different types of cancer [58].

To our knowledge, so far no one has determined the expression level of all eight genes from the *BIRC* family in patients with TNBC, therefore, the aim of the study was to determine the expression level of *BIRC1*, *BIRC2*, *BIRC3*, *BIRC4*, *BIRC5*, *BIRC6*, *BIRC7*, *BIRC8* genes in patients diagnosed with TNBC and to compare the obtained results with clinical data in order to determine the role of the discussed genes as prognostic factors of TNBC. Using bioinformatics tools (Ualcan and The Breast Cancer Gene-Expression Miner v4.5 (bc-GenExMiner v4.5)), the obtained data was compared with the data in the Cancer Genome Atlas (TCGA) database.

## 2. Results

### 2.1. Level of Expression of the BIRC Family Genes in Breast Cancer Tissue of Patients with TNBC Compared to Normal Tissue Surrounding the Tumor. Comparison of the Obtained Results with the Bioinformatic Analysis of Data Obtained from TCGA

**Figure 1:**
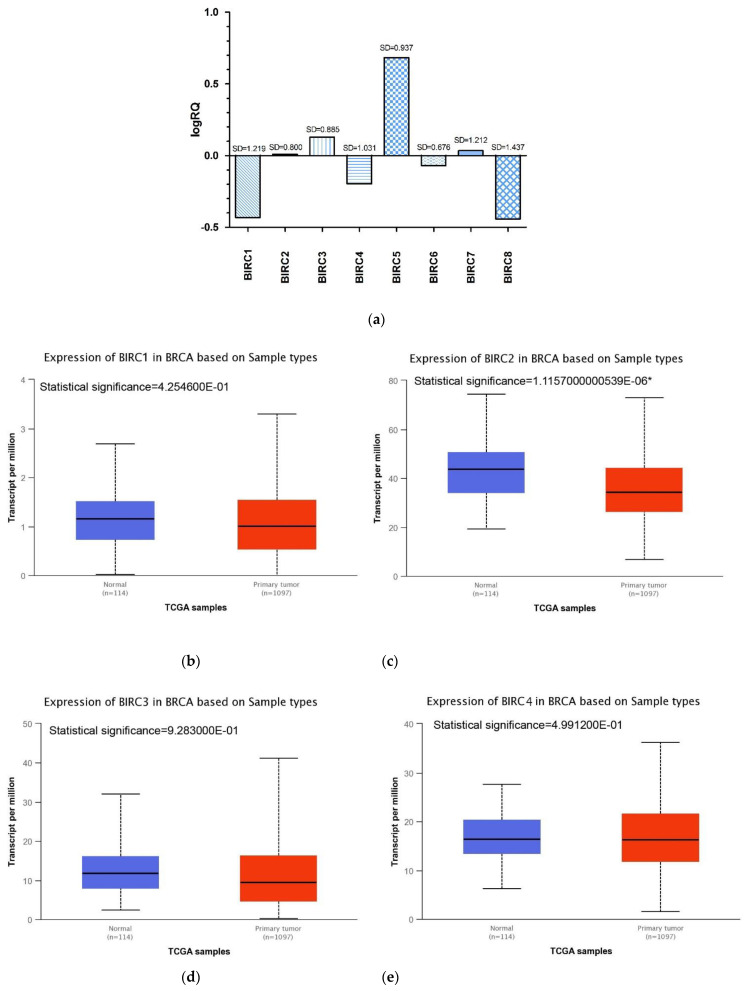

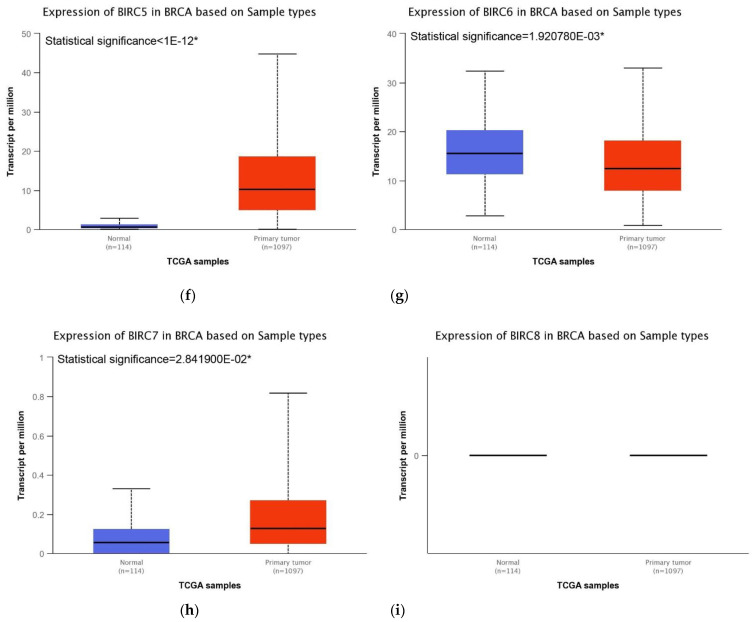
Average expression level of the tested *BIRC* family genes in patients with TNBC (**a**) and comparison of *BIRC1* (**b**), *BIRC2* (**c**), *BIRC3* (**d**), *BIRC4* (**e**), *BIRC5* (**f**), *BIRC6* (**g**), *BIRC7* (**h**) gene expression in normal tissues and breast cancer patients obtained using the Ualcan online tool. No information was found in the TCGA database on the level of *BIRC8* gene expression (**i**) in patients with breast cancer (*statistically significant).

Table 1 presents descriptive statistics for eight genes from the *BIRC* family. The highest mean value of expression among the studied genes was shown by *BIRC5* gene (M = 0.683783), while the lowest mean value of expression was recorded for *BIRC8* gene (M = −0.442143). The *BIRC2*, *BIRC3*, *BIRC5*, *BIRC7* genes showed an average increase in the expression level in the test sample as compared to the control, while the *BIRC1, BIRC4, BIRC6, BIRC8* genes showed a decreased expression level (Table 1, Figure 1a). 

The experimental data was compared with the data obtained as a result of the bioinformatic analysis of the TCGA database with the use of the Ualcan online tool. Bioinformatic analysis confirmed statistically significant increased levels of expression of the *BIRC5* and *BIRC7* genes and the decreased level of the *BIRC6* gene in patients with BC compared to the control group (Figure 1). The expression level of the *BIRC2* gene obtained as a result of the analysis of data from the TCGA database differed from the expression values of the discussed genes obtained experimentally (Figure 1). In the case of other genes from the BIRC family, bioinformatic analysis did not show statistically significant differences in the level of gene expression in the control group and breast cancer patients (Table S1, Figure 1).

The statistical analysis of the expression level of BIRC genes in patients with TNBC and in patients without TNBC obtained with the Breast Cancer Gene-Expression Miner v4.5 online tool showed statistically significant differences in the expression level of *BIRC1*, *BIRC2*, *BIRC3*, *BIRC4*, and *BIRC5* genes in patients from TCGA database depending on the type of molecular breast cancer. There was statistically significantly higher expression level of *BIRC2* (*p* < 0.0001)*, BIRC3* (*p* < 0.0001), and *BIRC5* genes (*p* < 0.0001) in TNBC patients compared to non-TNBC patients, and statistically significantly lower *BIRC1* (*p* = 0.0011) and *BIRC4* gene expression levels (*p* < 0.0001) in patients with triple negative breast cancer compared to other BC patients (Figure S1).

The above analysis justifies the purposefulness of the performed determination of the expression levels of BIRC genes in patients with triple-negative breast cancer and the correlation of the obtained expression values with clinical data due to the heterogeneous expression profile of the genes in question depending on the expression of ER, PR, and HER2 receptors in breast cancer.

### 2.2. The Relationships between the Expression Levels of the Examined Genes in TNBC. Comparison of the Obtained Results with the Bioinformatic Analysis of Data Obtained from TCGA

**Figure 2:**
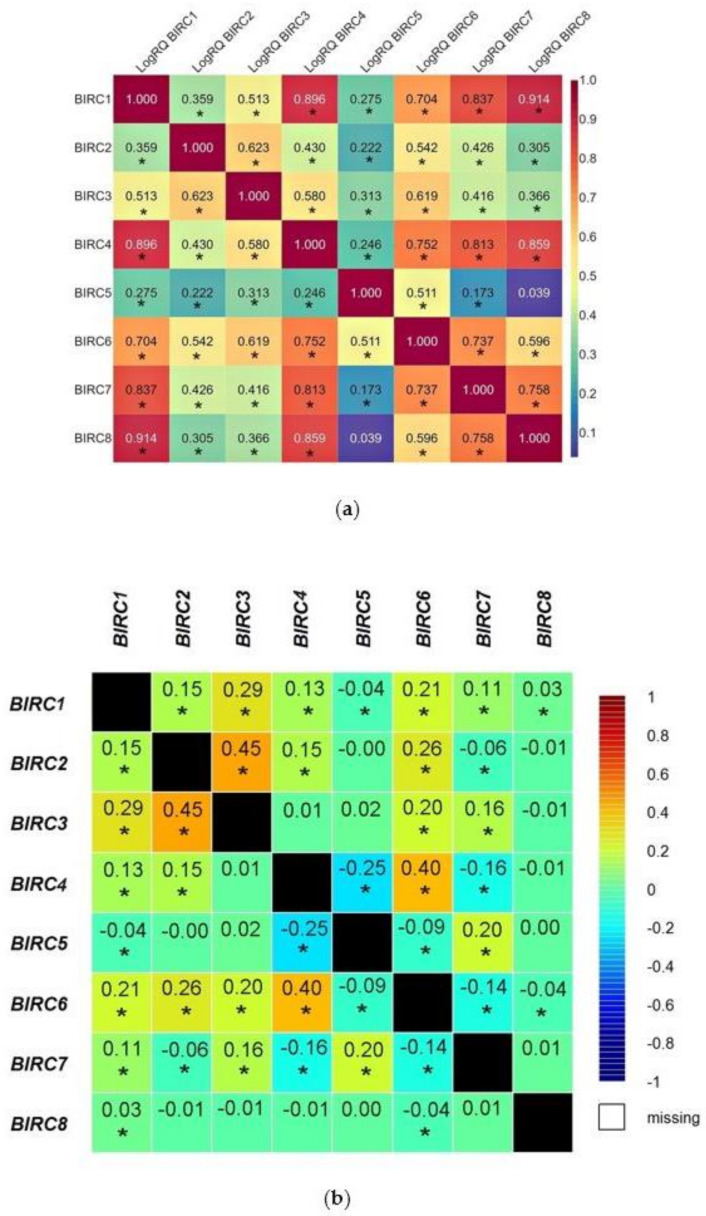
The results of the correlation analysis of the expression values of the BIRC family genes in TNBC (**a**) and the correlation analysis of the expression values of the BIRC family genes in BC obtained using the Breast Cancer Gene-Expression Miner v4.5 online tool (*r*-Pearson correlation coefficient) (**b**) (* statistically significant).

The analysis showed that almost all studied genes combine statistically significant positive correlations—the exception was one, statistically insignificant correlation of *BIRC5* with the *BIRC8* (*r* = 0.039) genes. The highest values of the correlation coefficients were found for the relationship between the *BIRC1* gene and the *BIRC8* (*r* = 0.914), *BIRC4* (*r* = 0.896) and *BIRC7* (*r* = 0.837) genes, the *BIRC4* gene with the *BIRC8* (*r* = 0.859) and the *BIRC7* (*r* = 0.813) genes. The lowest values of the correlation coefficient were found for most correlations of the *BIRC5* gene with the *BIRC7* (*r* = 0.173), *BIRC2* (*r* = 0.222), *BIRC4* (*r* = 0.246), and *BIRC1* (*r* = 0.275) genes (Figure 2a).

The experimental data was compared with the data obtained as a result of the bioinformatic analysis of the TCGA database with the use of the Breast Cancer Gene-Expression Miner v4.5 online tool. Bioinformatics analysis confirmed statistically significant positive correlations between the *BIRC1* gene and the *BIRC2* (*r* = 0.15), *BIRC3* (*r* = 0.29), *BIRC4* (*r* = 0.13), *BIRC6* (*r* = 0.21), *BIRC7* (*r* = 0.11), *BIRC8* (*r* = 0.03) genes; the *BIRC2* gene and the *BIRC3* (*r* = 0.45), *BIRC4* (*r* = 0.15), *BIRC6* (*r* = 0.26) genes; the *BIRC3* gene with the *BIRC6* (*r* = 0.2) and *BIRC7* (*r* = 0.16) genes; the *BIRC4* gene with the *BIRC6* gene (*r* = 0.4), the *BIRC5* gene with the *BIRC7* gene (*r* = 0.2) (Figure 2b).

### 2.3. The Analysis of the Dependence between Gene Expression and Clinical Data. Comparison of the Obtained Results with the Bioinformatic Analysis of Data Obtained from TCGA

The relationships between the expression level of the BIRC family genes and the patient age, lymphovascular invasion, invasion of the fat tissue, tumor size, metastases to the lymph nodes and SBR grade were analyzed.

#### 2.3.1. Age

**Figure 3:**
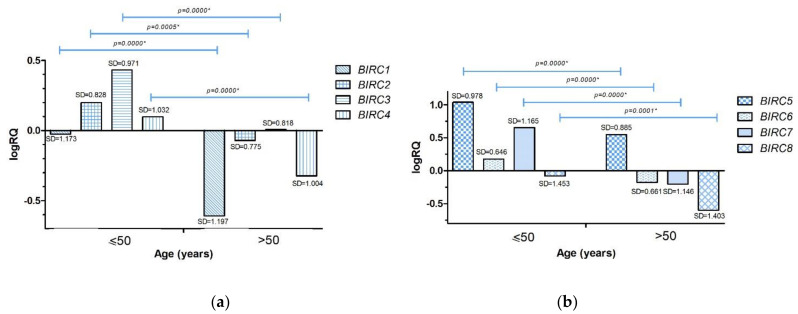
Mean expression level (logRQ) of the *BIRC1*, *BIRC2*, *BIRC3*, *BIRC4* (**a**), *BIRC5*, *BIRC6*, *BIRC7*, *BIRC8* (**b**) genes in breast cancer tissue in groups depending on the patients age (≤50 years, >50 years). * The significance level of the U Mann–Whitney test.

The analysis carried out with the U Mann–Whitney test showed that the level of expression all the tested BIRC family genes was statistically significantly higher in women with triple negative breast cancer diagnosed before the age of 50 (*p* < 0.05; the exact significance level was indicated in the charts) (Table S2, Figure 3a,b).

The experimental data was compared with the data obtained as a result of the bioinformatic analysis of the TCGA database with the use of the Breast Cancer Gene-Expression Miner v4.5 online tool. Bioinformatics analysis of publicly available data from the TCGA database confirmed statistically significant higher levels of *BIRC2* (*p* = 0.0213), *BIRC3* (*p* = 0.0029), *BIRC5* (*p* = 0.0040) gene expression in breast cancer patients under 51 years of age. In the case of the *BIRC4* gene (*p* = 0.0110), a statistically significant reduced level of expression was found in patients over 51 years of age (Figure S2).

#### 2.3.2. Lymphovascular Invasion

**Figure 4:**
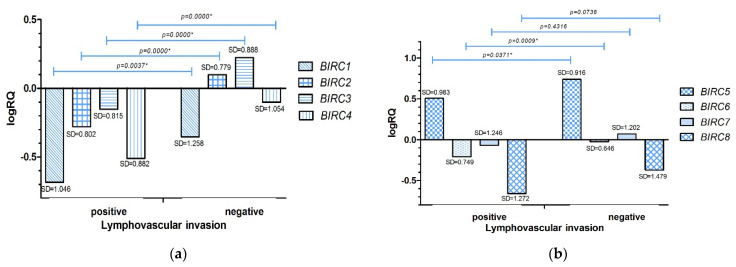
Mean expression level (logRQ) of the *BIRC1*, *BIRC2*, *BIRC3*, *BIRC4* (**a**), *BIRC5*, *BIRC6*, *BIRC7*, *BIRC8* (**b**) genes in breast cancer tissue in groups depending on the lymphovascular invasion. * The significance level of the U Mann–Whitney test.

The analysis carried out with the U Mann–Whitney test showed that the expression level of the tested genes *BIRC1* (*p* = 0.0004), *BIRC2* (*p* = 0.0000), *BIRC3* (*p* = 0.0000), *BIRC4* (*p* = 0.0000), *BIRC5* (*p* = 0.0372), *BIRC6* (*p* = 0.0009), was statistically significantly higher in women without lymphovascular invasion. In the case of the *BIRC7* (*p* = 0.4316) and *BIRC8* (*p* = 0.0738) genes, the difference was not statistically significant (Table S2, Figure 4a,b).

The experimental data was compared with the data obtained as a result of the bioinformatic analysis of the TCGA database with the use of the Breast Cancer Gene-Expression Miner v4.5 online tool. Bioinformatics analysis of publicly available data from the TCGA database demonstrated in contrast to experimental data statistically significantly higher levels of *BIRC4* gene expression in breast cancer patients with lymphovascular invasion (*p* = 0.0010). In the case of the other genes, no statistically significant differences in dependence on lymphovascular invasion were found (*p* > 0.05*)* (Figure S3).

#### 2.3.3. Cancer Cell Invasion of the Fat Tissue

**Figure 5:**
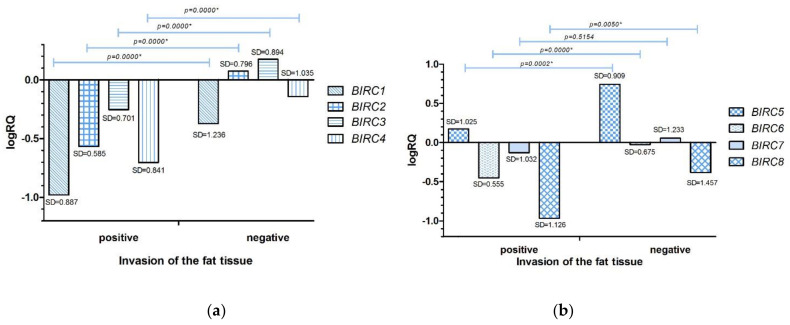
Mean expression level (logRQ) of the *BIRC1*, *BIRC2*, *BIRC3*, *BIRC4* (**a**), *BIRC5*, *BIRC6*, *BIRC7*, *BIRC8* (**b**) genes in breast cancer tissue in groups depending on the cancer cell invasion of the fat tissue. * The significance level of the U Mann–Whitney test.

Statistical analysis carried out with the use of the U Mann–Whitney test showed a statistically significantly higher level of expression of the *BIRC1* (*p* = 0.0000), *BIRC2* (*p* = 0.0000), *BIRC3* (*p* = 0.0000), *BIRC4* (*p* = 0.0000), *BIRC5* (*p* = 0.0003), *BIRC6* (*p* = 0.0000), *BIRC8* (*p* = 0.0000) genes in patients with TNBC who did not have cancer cell invasion of the fat tissue. In the case of the *BIRC7* (*p* = 0.5154) gene, the difference was not statistically significant (Table S3, Figure 5a,b).

The obtained data were not compared with the results of the bioinformatic analysis of the TCGA database due to the lack of information a given clinical parameter in the database described.

#### 2.3.4. Tumor Size

**Figure 6:**
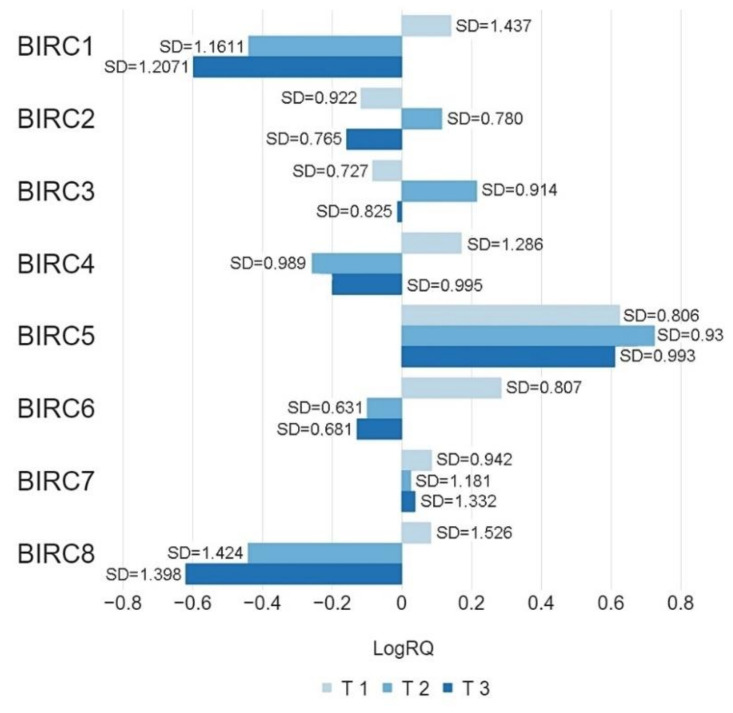
Mean values of expression of the studied genes in patients classified into T1, T2, T3 groups by tumor size. Metastases to the regional lymph nodes.

The conducted analysis showed statistically significant differences in the *p*-values of *BIRC1*, *BIRC6*, and *BIRC8* genes expression in patients with a primary tumor size ≤ 20 mm (T1) and patients with primary tumor size > 20 mm but ≤ 50 mm (T2) as well as patients from the T1 group and patients with whose primary tumor size was greater than 50 mm (T3), and there was no statistically significant difference in the level of expression of this gene in patients classified as T2 and T3 taking into account the size of the tumor. In the case of the *BIRC2* gene, there was a statistically significant difference in the value of gene expression in patients from the T2 and T3 groups, and no statistically significant differentiation for the patients from the T1 and T2, T1 and T3 groups. In the case of the *BIRC3* gene, there was a statistically significant difference in the values of gene expression in patients from the T1 and T2, T2 and T3 groups, but not in the T1 and T3 patients. The analysis showed a statistically significant differentiation in the *BIRC4* gene expression *p*-values in patients from the T1 and T2 group and no significant differentiation in patients from the T1 and T3, T2 and T3 groups. In the case of the *BIRC5* and *BIRC7* genes, the analysis did not show any statistically significant differences in patients from any of the groups (Table 2, Figure 6).

The obtained data were not compared with the results of the bioinformatic analysis of the TCGA database due to the lack of information a given clinical parameter in the database described.

**Figure 7:**
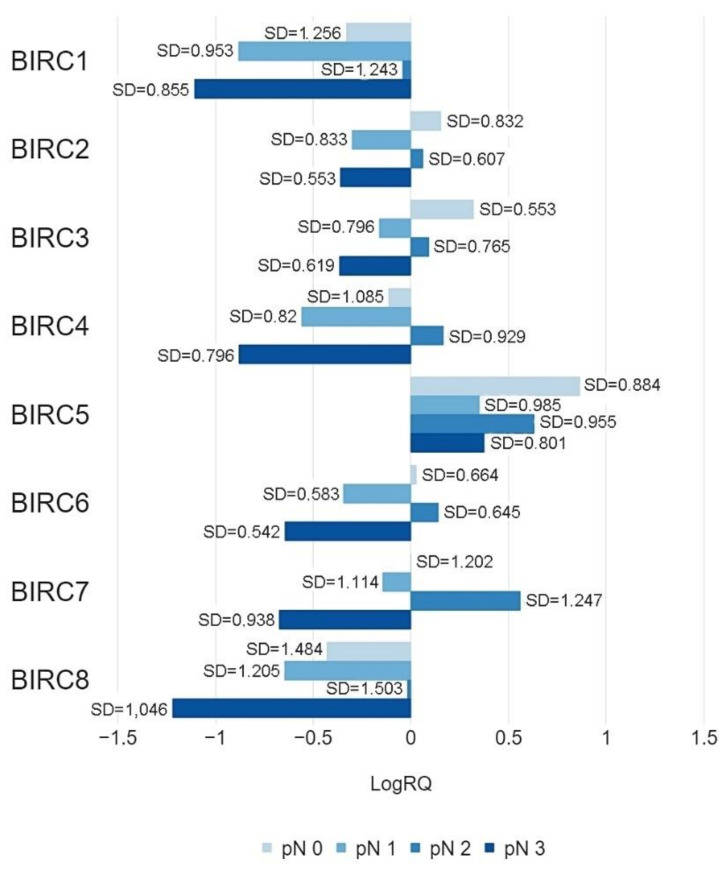
Mean values of expression of the studied genes in patients classified into pN0, pN1, pN2, pN3 groups by the metastases to the regional lymph nodes.

The analysis showed statistically significant differences in the *BIRC1*, *BIRC2*, *BIRC3* genes expression values in patients with no metastases to the regional lymph nodes (pN0) and patients with identified micrometastases or metastases in 1–3 axillary lymph nodes (pN1), patients from the pN0 group and patients with metastases in 10 or more axillary lymph nodes (pN3), patients in the pN1 group and patients with metastases in 4–9 axillary lymph nodes (pN2), patients in the pN2 and pN3 groups. There is no statistically significant difference in the level of expression of this gene in patients from the pN0 and pN2, pN1 and pN3 groups. A statistically significant differentiation of the *BIRC4* gene expression values was demonstrated in the pN0 and pN1, pN0 and pN2, pN0 and pN3, pN1 and pN2, pN2 and pN3 groups. There was no statistically significant difference in the level of expression of this gene in pN1 and pN3 patients. The *BIRC5* gene expression values were statistically significantly different in the pN0 and pN1, pN0 and pN3 groups. There was no statistically significant difference in the expression level of this gene in patients from the pN0 and pN2, pN1 and pN2, pN1 and pN3, pN2 and pN3 groups. Statistically significant differentiation of *BIRC6* gene expression levels were obtained for patients from the pN0 and pN1, pN0 and pN3, pN1 and pN2, pN1 and pN3, pN2 and pN3 groups. There was no statistically significant difference in the level of expression of this gene in pN0 and pN2 patients. The conducted analysis showed a statistically significant difference in the expression of the *BIRC7* gene in patients from the pN0 and pN2, pN1 and pN2, pN2 and pN3 groups, and no statistically significant difference in the expression level of this gene in patients from the pN0 and pN1, pN0 and pN3 groups, or pN1 and pN3. A statistically significant differentiation of the *BIRC8* gene expression values was demonstrated in the pN0 and pN3, pN1 and pN2, pN2 and pN3 groups. There was no statistically significant difference in the level of expression of this gene in patients from the pN0 and pN1, pN0 and pN2, pN1 and pN3 groups (Table 3 and Figure 7).

The experimental data was compared with the data obtained as a result of the bioinformatic analysis of the TCGA database with the use of the Ualcan online tool. Bioinformatic analysis confirmed a statistically significant difference in the level of expression of *BIRC3* gene in patients with BC from the pN0 and pN1, pN0 and pN3 groups, *BIRC4* gene in patients from pN0 and pN1, pN0 and pN2, pN2 and pN3, *BIRC5* gene in patients from pN0 and pN3 (Table S4, Figure S4).

#### 2.3.5. The Scarff-Bloom and Richardson (SBR) Grading System

**Figure 8:**
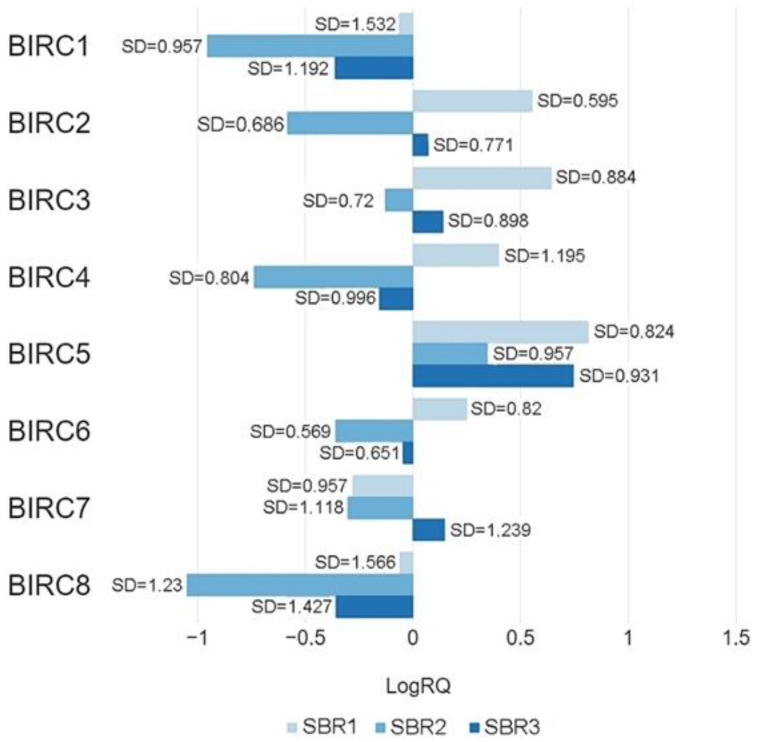
Mean values of expression of the studied genes in patients classified into SBR1, SBR2, SBR3 groups (criterion—tumor grade according to the Scarff-Bloom and Richardson (SBR) grading system).

The H Kruskal–Wallis test with an analysis of multiple comparisons showed statistically significant differences in the expression values of the studied genes between patients classified into three groups (criterion—tumor grade according to the Scarff-Bloom and Richardson (SBR) grading system). Analysis showed a statistically significant difference in the expression value of the *BIRC1*, *BIRC5,* and *BIRC8* genes in patients in the SBR1 and SBR2, SBR2, and SBR3 groups, and no statistically significant difference in the expression level of this gene in patients in the SBR1 and SBR3 groups. In the case of the *BIRC2*, *BIRC3*, *BIRC4*, and *BIRC6* genes, there was a statistically significant differences in the gene expression values in patients in the SBR1 and SBR2, SBR1 and SBR3, SBR2 and SBR3 groups. In the case of the *BIRC7* gene, the analysis did not show any statistical significance differentiation in the expression level between patients in the SBR1, SBR2, and SBR3 groups (Table 4, Figure 8).

The obtained data were not compared with the results of the bioinformatic analysis of the TCGA database due to the lack of information a given clinical parameter in the database described.

### 2.4. Effect of the Expression Values of the BIRC Family Genes on Breast Cancer Patients Overall Survival

**Figure 9:**
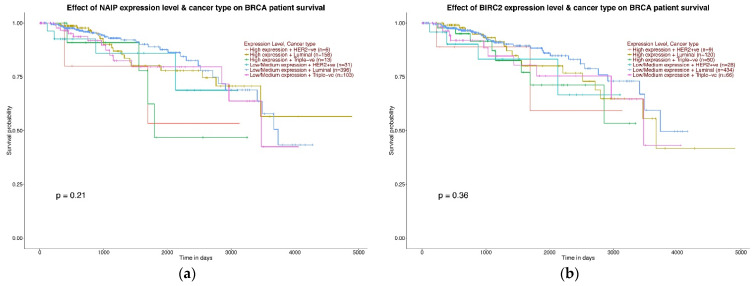

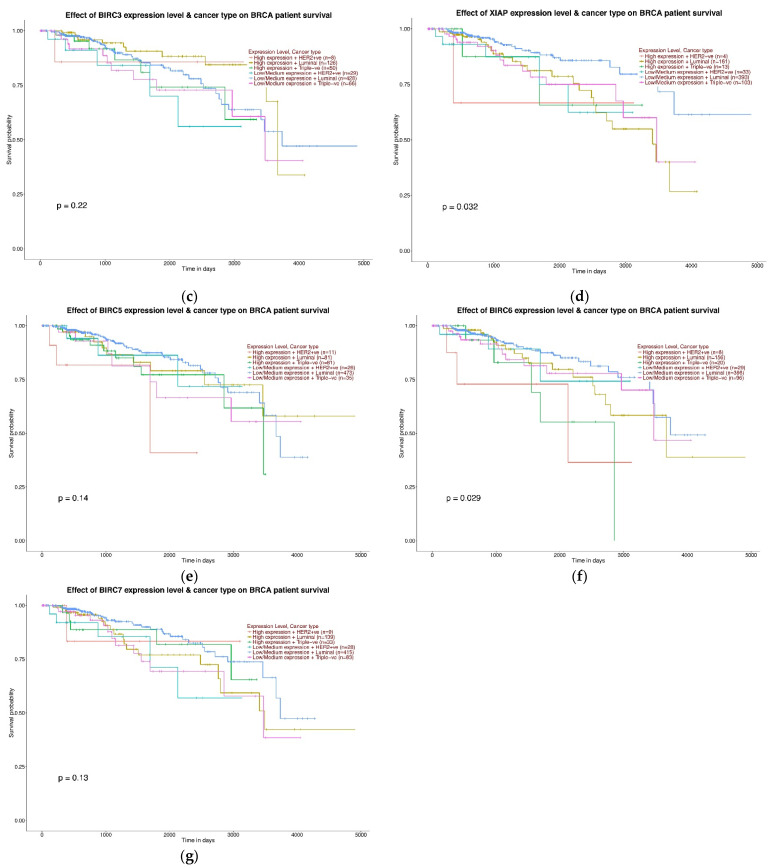
Survival curves (from the Kaplan–Meier Plotter) obtained using the Ualcan online tool representing evaluating the prognostic significance of *BIRC1 (NAIP*) (**a**), *BIRC2* (**b**), *BIRC3* (**c**), *BIRC4 (XIAP)* (**d**), *BIRC5* (**e**), *BIRC6* (**f**), *BIRC7* (**g**) on patients with different molecular types of breast cancer.

The prognostic value of *BIRC* family genes in patients with TNBC was investigated using the Kaplan–Meier Plotter. A statistically significant correlation was found between the elevated level of the *BIRC4* (*p* = 0.032) and *BIRC6* genes (*p* = 0.029) and the shorter overall survival (OS) of patients with TNBC (Figure 9). For the other studied genes, no statistical significance was found between the expression value and OS (*p >* 0.05) (Figure 9). The *BIRC8* gene was not analyzed due to the lack of data in the TCGA database on the level of expression of this gene and OS.

## 3. Discussion

Based on the analysis of articles in Pubmed and Web of Science databases, it can be concluded that the results of the expression level of *BIRC* family genes in the triple negative breast cancer model, published so far, mainly concern in vitro studies on breast cancer cell lines. In the case of studies performed with the use of human tissues, the authors often do not take into account the division of breast cancer into molecular subtypes or do not perform the determination of the level of all the genes in question in the same patients. Some of the published articles also concern the statistical analysis of the expression level of the genes in question from global databases, which creates the risk of analyzing data obtained by various research techniques or heterogeneous criteria for qualifying to the study group. So far, published studies have focused primarily on the role of the *BIRC5* gene in breast cancer, and the clinical significance of other *BIRC* genes has not been thoroughly investigated.

In our work, we present data on the expression levels of the all (eight) BIRC family genes in patients who were qualified for the study according to specific guidelines, and the study methodology was standardized.

The *BIRC* family genes encode for IAP proteins that are inhibitors of apoptosis. IAPs regulate the process of apoptosis by participating in the external and internal pathways and in the executive phase of apoptosis [58,59,60]. The dysregulation of the expression levels of the genes in question in cancer tissue as compared to normal tissue suggests that the apoptosis process in cancer cells was disturbed, which may be associated with the development of cancer.

We observed that the *BIRC2, BIRC3, BIRC5,* and *BIRC7* genes showed the increased levels of expression in tumor tissue compared to normal tissue, while in the case of the *BIRC1*, *BIRC4*, *BIRC6*, and *BIRC8* genes, we saw the decreased expression levels.

The *BIRC5* gene encoding the survivin protein showed the highest level of expression (Table 1, Figure 1a).

Comparing the expression level of the studied genes with the clinical data, we found that in patients diagnosed with breast cancer under the age of 50, the expression levels of all studied genes were higher compared to patients diagnosed after the age of 50 (Figure 3a,b). We observed that in patients with invasion of neoplastic cells into lymphatic vessels (Figure 4a,b) and fat tissue (Figure 5a,b), the expression levels of *BIRC* family genes were lower compared to patients in whom these features were not noted.

Unlike normal tissue, fat tissue cells (adipocytes) are in direct contact with cancer cells. Adipocytes supply tumor cells with lipids, which are a source of energy, and adipokines play a significant role in tumor expansion. Cancer cell interactions with fat tissue cells have been shown to support the progression of breast cancer [61,62]. Analyzing our results, it can be assumed that the decreased level of *BIRC* genes in patients with cancer cells invasion into fat tissue may be associated with the inhibition of apoptosis of breast cancer on the other pathways in which IAPs do not participate.

In most of the studied genes, statistically significant differences were also found in the values of expression in patients without regional lymph node metastases and in patients with diagnosed micrometastases or metastases to axillary lymph nodes (Figure 7a,b). In the case of patients with metastases in 10 or more axillary lymph nodes, the expression level of all tested genes was the lowest compared to other groups of patients. Higher expression levels of the *BIRC2*, *BIRC3*, and *BIRC5* genes were observed in patients without regional lymph node metastases compared to patients who had metastases. Statistically significant differences in gene expression were also noted in patients classified into three groups depending on tumor size (Table 3, Figure 6) or on the basis of tumor grade according to the Scarff-Bloom and Richardson (SBR) grading system. The lowest levels of expression of the BIRC family genes were observed in patients from the SBR2 group, while the highest levels were observed in patients from the SBR1 group (except the *BIRC7* gene) (Table 4, Figure 8). For all clinical features included in the study, the expression levels of the studied genes were highly diversified depending on the criterion of patient allocation to groups.

Our results partially overlap with the data published by Jian-bo Dai et al. who showed that the *BIRC5* gene was more strongly expressed in breast cancer patients compared to healthy controls. They found no significant difference in the level of *BIRC5* gene expression between the groups aged ≤51 and >51 years. In the results presented by the researchers, the high level of *BIRC5* was associated with a more advanced degree of SBR. They also showed no significant difference in the expression of the gene in question in the presence or absence of lymph node metastases in patients. The differences compared to our results may result from the fact that researchers did not take into account the division into molecular subtypes of breast cancer [63].

Wang Chen and coworkers obtained similar results to ours. Researchers showed that the *BIRC5* gene was highly expressed in TNBC, and *BIRC5* repression allowed the reduction of the proliferation of human breast cancer lines [64].

Baoai Han et al. found that the *BIRC5* gene was more strongly expressed in TNBC patients compared to other molecular breast cancer subtypes and control [65]. According to the available data, it can be assumed that the *BIRC5* gene may be a factor involved in tumor formation and the processes of disease invasion and progression [66]. Literature data indicate that high levels of the *BIRC5* gene in breast cancer patients may be associated with resistance to treatment with paclitaxel, doxorubicin, and gemcitabine [67,68]. Several studies have shown an association between *BIRC5* overexpression and survival in breast cancer patients [69,70,71,72]. The role of the *BIRC5* gene has also been identified as a prognostic factor for breast cancer patients without a pathological complete response (pCR) after neoadjuvant chemotherapy [73]. Based on the above data, it can be concluded that the *BIRC5* gene and the survivin it encodes are highly expressed in breast cancer cells as opposed to normal tissue. It can be assumed that an increase in the expression of this gene occurs during the early stage of cancer transformation, when the balance between proliferation and cell death is disturbed. Therefore, the *BIRC5* gene and survivin may be an effective therapeutic target for breast cancer, including TNBC.

Our data on the increased expression of the *BIRC7* gene in breast cancer compared to normal tissue is consistent with the results obtained by Fan Li et al. They showed that the level of Livin expression was higher in breast cancer with a higher histopathological malignancy, which was confirmed by the data we obtained (Table 4, Figure 8). Contrary to our results, the researchers found that Livin expression increased with the increase in lymph node metastases and was not closely related to age. The discrepancy in the obtained results may be caused by the application of other criteria for assigning patients to specific groups [50]. Livin has also been found to play a significant role in the resistance of breast cancer cells to transtuzumab treatment through the AKT and ERK1/2 pathways [51]. Therefore, livin, like survivin, may be an important target of anticancer therapy.

Bioinformatic analysis of the BIRC family gene expression results obtained from The Cancer Genome Atlas (TCGA) showed, similarly to our study, an increase in the expression level of the *BIRC5* and *BIRC7* genes, and a decrease in the expression level of the *BIRC1*, *BIRC4*, and *BIRC6* genes in breast cancer [15]. In their studies, they compared the expression level of *BIRC* family genes with the tumor stage. Stage of the cancer was defined as the stage of the tumor and the extent to which it spread throughout the body, which does not provide specific information on all clinical features of breast cancer that were taken into account. This makes it impossible to compare this data with the results obtained by us. Our clinical criteria included detailed information on the clinical and pathological features of the tumor, which were correlated with the level of gene expression. In the cited paper, the authors have shown that higher expression of the *BIRC5* and *BIRC7* genes is associated with higher tumor staging, and higher expression of the *BIRC5* gene was associated with worse survival across breast cancers [15].

The discrepancies in some of the results presented in the work were obtained as a result of our bioinformatics analysis using the Ualcan and the Breast Cancer Gene-Expression Miner v4.5 online tools compared to the levels of expression of *BIRC* genes and correlation with clinical data result from the comparison of experimental data of TNBC patients with data for patients with breast cancer without division into molecular subtypes (obtained from the TCGA database). Bioinformatics analysis also included a much larger group of patients with breast cancer compared to our study group. The methodology of conducting experiments resulting in the obtaining of the data presented in the TCGA database is not fully homogeneous with the research methods used by us. However, despite the described limitations of bioinformatics analysis, a large part of the results obtained by us are consistent with the data obtained using online tools.

We identified, to our knowledge for the first time, the expression levels of all genes from the *BIRC* family in the neoplastic tissue of a tumor collected from patients diagnosed with triple negative breast cancer, not undergoing neoadjuvant chemotherapy. The data presented provide the first information on the correlation of the expression level of *BIRC* genes with clinical data and the relationships between the expression level of the examined genes in TNBC. In addition, it can be concluded that the level of expression of *BIRC* genes may be related to the stage of cancer and be one of the determinants of the severity of the course of breast cancer and the potential for survival, taking into account clinical prognostic factors. However, the confirmation of the presented hypothesis about the role of *BIRC* genes in TNBC still requires detailed experiments.

## 4. Materials and Methods

### 4.1. Characteristics of the Study Group

The research was conducted in accordance with the Declaration of Helsinki. The study was approved by the Ethics Committee at Medical University of Lublin; decision number: KE-0254/216/2014. All patients gave their informed consent to participate in the research. The oncologist classified the patients (women) for research according to specific guidelines. In the project were included 30 patients diagnosed with triple negative breast cancer. Patients were treated at the Oncology Center in Lublin. Patients did not express the ER receptor, PR and HER2 in cancer tissue. ER, PR, and HER2 expression was determined using immunohistochemistry (IHC). The test material used at IHC were tissue fragments fixed in buffered formalin and embedded in paraffin. The expression levels of ER, PR, and HER2 receptors were determined using standard procedure [74]. The expression of ER, PR, and HER2 receptors was assessed by two independent pathologists. The age of patients that qualified for the study ranged from 33 to 79 years (57.93 ± 11.72—mean ± SD). The criterion for excluding patients from the project was the presence of other diseases. The patients included in the study did not use any medications chronically. The patients did not indicate any family history of cancer diseases. Clinical data: age, sex, familial history of cancer, lymphovascular invasion, invasion of the fat tissue, primary tumor size, metastases to the regional lymph nodes. SBR grades were obtained on the basis of a review of clinical documentation and pathological data. The SBR grades according to the Scarff-Bloom-Richardson scale were assessed by two independent pathologists from the Oncology Center in Lublin according to the standard criteria described in the literature [75]. Patients enrolled in the study were not subjected to neoadjuvant chemotherapy. Detailed information on the characteristics of the patients is provided in Table 5.

### 4.2. Preparation of the Material for RNA Isolation

During the surgical procedure, from patients a tumor tissue fragment (test sample) and a tissue fragment surrounding the tumor (control sample) were collected. The collected tissues were examined by pathologists to confirm their qualification for the study and control groups. Maintaining sterile conditions, collected tissues were placed in sterile tubes with RNA-later solution (Invitrogen, Carlsbad, CA, USA) and stored at −20 °C for RNA analysis.

### 4.3. Tissue Homogenization

The homogenization of the collected tissues was carried out using the Precellys 24 homogenizer (Bertin-Instruments, Montigny-le-Bretonneux, France) with the option of cooling Cryolys, enabling work with thermosensitive molecules. Tissue disintegration was achieved using a disintegrating material in the form of stainless-steel beads (TK Biotech, Warsaw, Poland) placed in homogenized biological material.

### 4.4. RNA Isolation and cDNA Reverse Transcription

Total RNA was isolated from collected tissues according to the protocol of the Single-step modified method of RNA isolation [78] using TRI Reagent Solution (Invitrogen, Carlsbad, CA, USA) and 1-Bromo-3-chloropropane (Sigma Aldrich, Saint Louis, MO, USA). The concentration and quality of RNA was determined using NanoDrop ND-1000 spectrophotometer (Thermo Fisher Scientific, Waltham, MA, USA). For all samples analyzed, A260/A280 ratio was between 1.8 and 2.0. Isolated RNA was stored at −80 °C until used. cDNA was synthesized using High Capacity cDNA Reverse Transcription Kit (Applied Biosystem, Foster City, CA, USA) according to the manufacturer’s protocol.

### 4.5. Gene Expression Analysis

This research was carried out by means of 384-well TaqMan™ Human Apoptosis Array (Applied Biosystems, Foster City, CA, USA) according to the manufacturer’s protocol. In research *ACTB*-Hs99999903_m1 was chosen as endogenous control. TLDA cards were run on a QuantStudio 12 K Flex Real-Time PCR System (Applied Biosystems, Foster City, CA, USA). Gene expression values were calculated using the comparative quantification method ΔΔCt with Expression Suite Software v 1.1. Gene expression in breast cancer tissues was compared with each normal tissue collected from patients enrolled in the study.

The relative expression level of the studied genes were determined using the comparative method (ΔΔCt, comparative). The basis of the comparative method is a mathematical model that allows to calculate the relative difference in the expression level of the tested gene between the test samples and the control sample. At the beginning of the analysis, the threshold cycles (Ct) of the amplification reaction of control and test genes are determined for the test samples and the control sample. In the next step, the differences between the values of Ct, PCR running on the template of the test gene and the control gene (∆Ct) are calculated [79].

Ct gene of BIRC family (sample) − Ct endogenous control (sample) = ΔCt sample

Ct gene of BIRC family (calibrator) − Ct endogenous control (calibrator) = ΔCt calibrator

Then ΔΔCt is calculated for each sample:

ΔΔCt = ΔCt (sample) − ΔCt (calibrator).

In the next step, the normalized value of the relative expression level of the test gene in the test sample compared to the calibrator is calculated using the formula:

RQ  =  2^–ΔΔCt^

In the analysis the expression levels of genes from the *BIRC* family *(BIRC1*-Hs01847653_s1 *BIRC2*-Hs00236911_m1 *BIRC3*-Hs00985031_g1 *BIRC4*-Hs00745222_s1 *BIRC5*-Hs00977611_g1, *BIRC6*-Hs00212288_m1, *BIRC7*-Hs00223374_m1, *BIRC8*-Hs01057786_s1) were included. The results were analyzed as logRQ values of gene expression [79].

### 4.6. Methods of Statistical Data Analysis

Statistica v.13.3, DisPlayr and GraphPad v.5.01 were used in the statistical analysis and graphic design (*p* < 0.05 was assumed statistically significant). U Mann–Whitney test, H Kruskall–Wallis test with multiple comparisons were used to calculate the differences in expression level between genes and r-Spearman coefficient with heatmap correlation matrix was used for correlation analysis.

The data contained in The Cancer Genome Atlas (TCGA) data were analyzed using the Internet sources Ualcan (http://ualcan.path.uab.edu/ (accessed on 11 February 2021)) [80] and the Breast Cancer Gene-Expression Miner v4.5 (bc-GenExMiner v4.5, http://bcgenex.centregauducheau.fr/BC-GEM (accessed on 11 February 2021)) [81,82].

## Figures and Tables

**Table 1:** Descriptive statistics for the expression values of the *BIRC* family genes in TNBC.

Gene	N Comparisons	Mean [logRQ]	SD [logRQ]	Median [logRQ]
*BIRC1*	740	−0.431581	1.218730	−0.386159
*BIRC2*	690	0.009995	0.800377	0.005800
*BIRC3*	554	0.129047	0.884598	0.192146
*BIRC4*	720	−0.197498	1.030632	−0.142367
*BIRC5*	522	0.683783	0.937065	0.648409
*BIRC6*	780	−0.069403	0.675635	−0.051101
*BIRC7*	277	0.034917	1.212470	0.026533
*BIRC8*	566	−0.442143	1.437147	−0.386170

**Table 2:** Descriptive statistics and the level of significance of the difference (H Kruskal–Wallis test with multiple comparison) in the expression of the studied genes in patients classified into T1, T2, T3 groups by tumor size.

Gene	T1		T2		T3		*p* for Multiple Comparison
	Mean	SD	Mean	SD	Mean	SD	
*BIRC1* [LogRQ]	0.141	1.4372	−0.439	1.1611	−0.597	1.2071	T1*T2 = 0.006674 T1*T3 = 0.000178 T2*T3 = 0.165
*BIRC2* [LogRQ]	−0.117	0.9221	0.115	0.78002	−0.158	0.7652	T1*T2 = 0.338 T1*T3 = 0.999 T2*T3 = 0.000211
*BIRC3* [LogRQ]	−0.083	0.7279	0.215	0.9147	−0.012	0.8259	T1*T2 = 0.025384 T1*T3 = 0.999 T2*T3 = 0.000439
*BIRC4* [LogRQ]	0.171	1.2865	−0.257	0.9891	−0.199	0.9956	T1*T2 = 0.041191 T1*T3 = 0.193 T2*T3 = 0.999
*BIRC5* [LogRQ]	0.625	0.8068	0.725	0.9314	0.611	0.9938	T1*T2 = 0.999 T1*T3 = 0.999 T2*T3 = 0.993
*BIRC6* [LogRQ]	0.285	0.8075	−0.099	0.6315	−0.128	0.6810	T1*T2 = 0.000648 T1*T3 = 0.000204 T2*T3 = 0.999
*BIRC7* [LogRQ]	0.086	0.9425	0.026	1.1819	0.038	1.3324	T1*T2 = 0.999 T1*T3 = 0.999 T2*T3 = 0.999
*BIRC8* [LogRQ]	0.083	1.5263	−0.4401	1.4244	−0.619	1.3987	T1*T2 = 0.049377 T1*T3 = 0.005931 T2*T3 = 0.478

**Table 3:** Descriptive statistics and the level of significance of the difference (H Kruskal–Wallis test with multiple comparison) in the expression of the studied genes in patients classified into pN0, pN1, pN2, pN3 groups by the metastases to the regional lymph nodes (identified by histological methods).

Gene	pN0		pN1		pN2		pN3		*p* for Multiple Comparison
	Mean	SD	Mean	SD	Mean	SD	Mean	SD	
*BIRC1* [LogRQ]	−0.329	1.2563	−0.8809	0.9536	−0.041	1.2437	−1.106	0.8559	pN0*pN1 = 0.000049 pN0*pN2 = 0.060788 pN0*pN3 = 0.000077 pN1*pN2 = 0.000000 pN1*pN3 = 0.924 pN2*pN3 = 0.000000
*BIRC2* [LogRQ]	0.153	0.8322	−0.3005	0.8336	0.061	0.6076	−0.3603	0.5536	pN0*pN1 = 0.000000 pN0*pN2 = 0.999 pN0*pN3 = 0.000124 pN1*pN2 = 0.001374 pN1*pN3 = 0.999 pN2*pN3 = 0.005638
*BIRC3* [LogRQ]	0.3202	0.9284	−0.1604	0.7963	0.092	0.7659	−0.364	0.6195	pN0*pN1 = 0.000000 pN0*pN2 = 0.188 pN0*pN3 = 0.000000 pN1*pN2 = 0.020712 pN1*pN3 = 0.203258 pN2*pN3 = 0.000174
*BIRC4* [LogRQ]	−0.113	1.0853	−0.558	0.8206	0.166	0.9291	−0.878	0.7969	pN0*pN1 = 0.000117 pN0*pN2 = 0.00258 pN0*pN3 = 0.000001 pN1*pN2 = 0.000000 pN1*pN3 = 0.120 pN2*pN3 = 0.000000
*BIRC5* [LogRQ]	0.864	0.8848	0.35001	0.9853	0.6305	0.9558	0.375	0.8014	pN0*pN1 = 0.000178 pN0*pN2 = 0.302 pN0*pN3 = 0.014555 pN1*pN2 = 0.598 pN1*pN3 = 0.999 pN2*pN3 = 0.770
*BIRC6* [LogRQ]	0.027	0.6647	−0.345	0.58303	0.1404	0.6459	−0.643	0.5425	pN0*pN1 = 0.000000 pN0*pN2 = 0.404 pN0*pN3 = 0.000000 pN1*pN2 = 0.000000 pN1*pN3 = 0.03835 pN2*pN3 = 0.000000
*BIRC7* [LogRQ]	−0.0006	1.20208	−0.144	1.1144	0.5592	1.24708	−0.673	0.93806	pN0*pN1 = 0.999 pN0*pN2 = 0.045489 pN0*pN3 = 0.121 pN1*pN2 = 0.032137 pN1*pN3 = 0.450 pN2*pN3 = 0.001058
*BIRC8* [LogRQ]	−0.429	1.4845	−0.646	1.2053	−0.017	1.5038	−1.218	1.0467	pN0*pN1 = 0.999 pN0*pN2 = 0.081 pN0*pN3 = 0. 012351 pN1*pN2 = 0.016192 pN1*pN3 = 0.167 pN2*pN3 = 0.000121

**Table 4:** Descriptive statistics and the levels of significance of the difference (H Kruskal–Wallis test with multiple comparison) in the expression of the studied genes in patients classified into SBR1, SBR2, SBR3 groups (criterion—tumor grade according to the Scarff-Bloom and Richardson (SBR) grading system).

Gene	SBR1		SBR2		SBR3		*p* for Multiple Comparison
	Mean	SD	Mean	SD	Mean	SD	
*BIRC1* [LogRQ]	−0.063	1.5324	−0.955	0.9574	−0.361	1.1927	SBR1*SBR2 = 0.000035 SBR1*SBR3 = 0.655 SBR2*SBR3= 0.000002
*BIRC2* [LogRQ]	0.553	0.5956	−0.583	0.6861	0.0709	0.7717	SBR1*SBR2 = 0.000000 SBR1*SBR3 = 0.000002 SBR2*SBR3 = 0.000000
*BIRC3* [LogRQ]	0.642	0.8845	−0.128	0.7207	0.1402	0.89809	SBR1*SBR2 = 0.000000 SBR1*SBR3 = 0.000027 SBR2*SBR3 = 0.000460
*BIRC4* [LogRQ]	0.397	1.1951	−0.737	0.804	−0.155	0.9969	SBR1*SBR2 = 0.000000 SBR1*SBR3 = 0.001191 SBR2*SBR3 = 0.000000
*BIRC5* [LogRQ]	0.815	0.8244	0.346	0.9573	0.745	0.9317	SBR1*SBR2 = 0.019157 SBR1*SBR3 = 0.999 SBR2*SBR3 = 0.003377
*BIRC6* [LogRQ]	0.2504	0.8205	−0.359	0.5693	−0.046	0.6519	SBR1*SBR2 = 0.000000 SBR1*SBR3 = 0.020894 SBR2*SBR3 = 0.000004
*BIRC7* [LogRQ]	−0.279	0.9576	−0.303	1.1185	0.147	1.2398	SBR1*SBR2 = 0.999 SBR1*SBR3 = 0.366 SBR2*SBR3 = 0.100
*BIRC8* [LogRQ]	−0.061	1.5664	−1.049	1.2303	−0.357	1.4276	SBR1*SBR2 = 0.000345 SBR1*SBR3 = 0.599 SBR2*SBR3 = 0.000115

**Table 5:** Characteristics of 30 patients with TNBC approved to the study. The histological type of breast cancer was determined according to the 4th edition Classification of Tumors—World Health Organization (WHO) for breast tumors [76]. Pathological tumor-node-metastasis (pTNM) was determined on the basis of 7th edition of the TNM classification of the American Joint Committee on Cancer (AJCC) [77]. The tumor grade was assessed according to the Scarff-Bloom and Richardson (SBR) grading system, Quantitative vascular invasion was determined with standard histological methods [75].

Characteristic	Patients with TNBC (*n* = 30)
**Age at diagnosis** ≤50 >50	8 (≈26.67%) 22 (≈73.33%)
**Familial history of cancer** Yes No	0 (0%) 30 (100%)
**Adjuvant chemotherapy** Yes No	0 (0%) 30 (100%)
**Gender**: Male Female	0 (0%) 30 (100%)
**Lymphovascular invasion** Yes No	10 (33.33%) 20 (66.67%)
**Invasion of the fat tissue** Yes No	5 (≈16.67%) 25 (≈83.33%)
**Tumor size** T1 T2 T3	3 (10%) 19 (≈63.33%) 8 (≈26.67%)
**Lymph nodes** N0 N1 N2 N3	17 (≈56.67%) 6 (20%) 5 (≈16.67%) 2 (≈6.67%)
**SBR grade** SBR1 SBR2 SBR3	3 (10%) 5 (≈16.67%) 22 (≈73.33%)

## Data Availability

The data that support the findings of this study are available from the corresponding author upon reasonable request.

## Supplementary Materials

Supplementary Materials can be found at https://www.mdpi.com/1422-0067/22/4/1820/s1.
