# Supplementary material for: The BIRC Family Genes Expression in Patients with Triple Negative Breast Cancer
Source: Int J Mol Sci. 2021 Feb 12;22(4):1820. doi: 10.3390/ijms22041820 (PMC7918547; doi:10.3390/ijms22041820)
Supplement: Supplementary file 1 [file ijms-22-01820-s001.zip › Table S1.docx]

Table S1. The BIRC family genes expression in normal tissues and breast cancer patients obtained from the Ualcan database - statistical significance.

| **Gene** | **Comparison** | **Statistical significance** |
| --- | --- | --- |
| BIRC1 (NAIP) | Normal-vs-Primary | 4.254600E-01 |
| BIRC2 | Normal-vs-Primary | **1.1157000000539E-06** |
| BIRC3 | Normal-vs-Primary | 9.283000E-01 |
| BIRC4 (XIAP) | Normal-vs-Primary | 4.991200E-01 |
| BIRC5 | Normal-vs-Primary | **<1E-12** |
| BIRC6 | Normal-vs-Primary | **1.920780E-03** |
| BIRC7 | Normal-vs-Primary | **2.841900E-02** |
| BIRC8 | Normal-vs-Primary | 5.239000E-01 |
