# Supplementary material for: The BIRC Family Genes Expression in Patients with Triple Negative Breast Cancer
Source: Int J Mol Sci. 2021 Feb 12;22(4):1820. doi: 10.3390/ijms22041820 (PMC7918547; doi:10.3390/ijms22041820)
Supplement: Supplementary file 1 [file ijms-22-01820-s001.zip › Table S2.docx]

Table S2. Descriptive statistics and the significance level of the difference (U Mann-Whitney test) in the expression of the studied genes in patients ≤50 and >50 years of age.

| Gene | ≤50 | | >50 | | p |
| --- | --- | --- | --- | --- | --- |
|  | M | SD | M | SD |  |
| LogRQ *BIRC1* | -0,025 | 1,1734 | -0,608 | 1,1965 | 0,000000 |
| LogRQ *BIRC2* | 0,199 | 0,8275 | -0,071 | 0,7751 | 0,000539 |
| LogRQ *BIRC3* | 0,431 | 0,9707 | 0,0071 | 0,8175 | 0,000000 |
| LogRQ *BIRC4* | 0,097 | 1,0322 | -0,323 | 1,0048 | 0,000000 |
| LogRQ *BIRC5* | 1,040 | 0,9779 | 0,547 | 0,8851 | 0,000000 |
| LogRQ *BIRC6* | 0,177 | 0,6459 | -0,175 | 0,6608 | 0,000000 |
| LogRQ *BIRC7* | 0,655 | 1,1654 | -0,203 | 1,1462 | 0,000001 |
| LogRQ *BIRC8* | -0,081 | 1,4528 | -0,599 | 1,4032 | 0,000092 |
