# Supplementary material for: The BIRC Family Genes Expression in Patients with Triple Negative Breast Cancer
Source: Int J Mol Sci. 2021 Feb 12;22(4):1820. doi: 10.3390/ijms22041820 (PMC7918547; doi:10.3390/ijms22041820)
Supplement: Supplementary file 1 [file ijms-22-01820-s001.zip › Table S3.docx]

Table S3. Descriptive statistics and the significance level of the difference (U Mann-Whitney test) in the expression of the studied genes in groups depending on the cancer cell invasion of the fat tissue.

| Gene | positive | | negative | | p |
| --- | --- | --- | --- | --- | --- |
|  | M | SD | M | SD |  |
| [LogRQ] BIRC1 | -0,978664 | 0,887275 | -0,371705 | 1,235557 | 0,000038 |
| [LogRQ] BIRC2 | -0,566632 | 0,585381 | 0,074065 | 0,795737 | 0,000000 |
| [LogRQ] BIRC3 | -0,253412 | 0,701453 | 0,174633 | 0,893672 | 0,000003 |
| [LogRQ] BIRC4 | -0,703513 | 0,841378 | -0,141274 | 1,034921 | 0,000003 |
| [LogRQ] BIRC5 | 0,173631 | 1,025074 | 0,742647 | 0,909289 | 0,000271 |
| [LogRQ] BIRC6 | -0,450212 | 0,555360 | -0,027091 | 0,674884 | 0,000000 |
| [LogRQ] BIRC7 | -0,131476 | 1,032150 | 0,055127 | 1,232878 | 0,515398 |
| [LogRQ] BIRC8 | -0,966482 | 1,126150 | -0,384568 | 1,456715 | 0,005045 |
