# Supplementary material for: The BIRC Family Genes Expression in Patients with Triple Negative Breast Cancer
Source: Int J Mol Sci. 2021 Feb 12;22(4):1820. doi: 10.3390/ijms22041820 (PMC7918547; doi:10.3390/ijms22041820)
Supplement: Supplementary file 1 [file ijms-22-01820-s001.zip › Table S4.docx]

**Table S4.** The level of significance of the difference in the expression of the studied genes in patients classified into pN0, pN1, pN2, pN3 groups by the metastases to the regional lymph nodes obtained from the Ualcan database

| **Gene** | **p for multiple comparison** |
| --- | --- |
| *BIRC1* | pN0*pN1= 5.934600E-01  pN0*pN2= 3.303800E-01  pN0*pN3= 6.377000E-01  pN1*pN2= 1.837600E-01  pN1*pN3= 5.023600E-01  pN2*pN3= 9.856800E-01 |
| *BIRC2* | pN0*pN1= 7.667800E-01  pN0*pN2= 5.475000E-01  pN0*pN3= 1.031200E-01  pN1*pN2= 4.218600E-01  pN1*pN3= 1.315630E-01  pN2*pN3= 6.039700E-02 |
| *BIRC3* | **pN0*pN1= 1.208030E-02**  pN0*pN2= 8.146200E-02  **pN0*pN3= 4.154200E-04**  pN1*pN2= 9.419000E-01  pN1*pN3= 1.372440E-01  pN2*pN3= 2.431800E-01 |
| *BIRC4* | **pN0*pN1= 7.941200E-03**  **pN0*pN2= 1.613870E-02**  pN0*pN3= 2.070800E-01  pN1*pN2= 5.943000E-01  **pN1*pN3= 3.819100E-03**  **pN2*pN3= 7.395300E-03** |
| *BIRC5* | pN0*pN1= 9.657400E-01  pN0*pN2= 6.433400E-01  **pN0*pN3= 4.015900E-02**  pN1*pN2= 6.870600E-01  pN1*pN3= 5.033500E-02  pN2*pN3= 1.167640E-01 |
| *BIRC6* | pN0*pN1= 2.669800E-01  pN0*pN2= 5.984000E-02  pN0*pN3= 4.151200E-01  pN1*pN2= 2.464000E-01  pN1*pN3= 1.429610E-01  **pN2*pN3= 4.384300E-02** |
| *BIRC7* | pN0*pN1= 8.150600E-01  pN0*pN2= 3.531400E-01  pN0*pN3= 3.761800E-01  pN1*pN2= 4.076200E-01  pN1*pN3= 4.393600E-01  pN2*pN3= 9.798800E-01 |
| *BIRC8* | pN0*pN1= 7.871600E-01  pN0*pN2= 6.351000E-01  pN0*pN3= N/A  pN1*pN2= 8.160200E-01  pN1*pN3= N/A  pN2*pN3= N/A |
