# Supplementary figures and images for: The BIRC Family Genes Expression in Patients with Triple Negative Breast Cancer
Source: Int J Mol Sci. 2021 Feb 12;22(4):1820. doi: 10.3390/ijms22041820 (PMC7918547; doi:10.3390/ijms22041820)

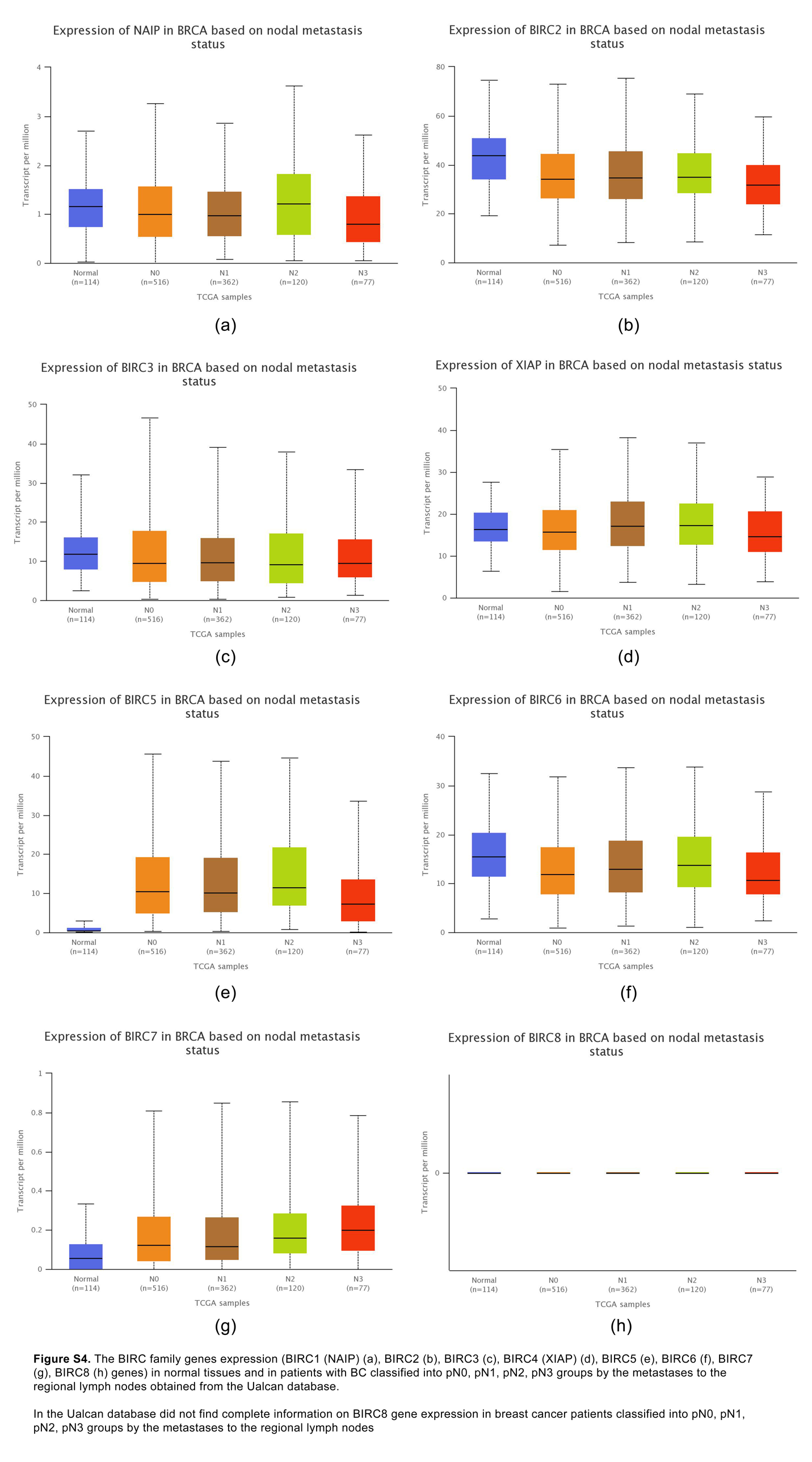

Supplement: Supplementary file 1 [file ijms-22-01820-s001.zip › Figure S4.jpg]
